# Device-level nonlinearity and temporal memory in optoelectronic reservoir computing

**DOI:** 10.1186/s40580-025-00522-0

**Published:** 2025-11-27

**Authors:** Won Woo Lee, Junhyung Cho, Jaehyun Hur, Hongseok Oh, Hocheon Yoo

**Affiliations:** 1 Department of Artificial Intelligence Semiconductor Engineering, Hanyang University, Seoul, 04763 Republic of Korea; 2 School of Chemical, Biological, and Battery Engineering, Gachon University, Gyeonggi, 13120 Republic of Korea; 3 Department of Physics and Integrative Institute of Basic Sciences, Soongsil University, Seoul, 06978 Republic of Korea; 4 Department of Electronic Engineering, Hanyang University, Seoul, 04763 Republic of Korea

**Keywords:** Physical reservoir computing, Optoelectronic reservoir computing, Nonlinear dynamics, Photodiodes, Memristors, Phototransistors

## Abstract

Reservoir computing (RC) has emerged as a promising computational paradigm for processing temporally correlated and nonlinear data with low training cost. Among various physical implementations, optoelectronic devices provide a unique opportunity to directly interface light with nonlinear dynamical systems, enriching the reservoir state space through device-intrinsic responses. Light can encode information in wavelength, intensity, and pulse duration, and stimulate multiple nodes in parallel with minimal delay or added power. Recent advances in photodiodes, optically modulated memristors, and phototransistors have revealed device-level pathways to enhance nonlinearity, temporal memory, and node diversity, moving beyond purely electrical control toward hybrid optical–electrical tuning. This review revisits these developments from a device physics perspective, highlighting mechanisms for multi-state generation, bidirectional synaptic weight modulation, and temporal response tailoring. We compare diverse excitation schemes, ranging from wavelength- and intensity-selective photocarrier modulation to con optical-assisted filament control and gate–light co-modulation. We also discuss their impact on reservoir performance in pattern recognition, time-series prediction, and dynamic signal processing. We connect material design, device architecture, and reservoir dynamics to outline emerging strategies for scaling optoelectronic RC. This review provides timely insights for researchers working at the intersection of device engineering and neuromorphic computing.

**Graphical Abstract:**

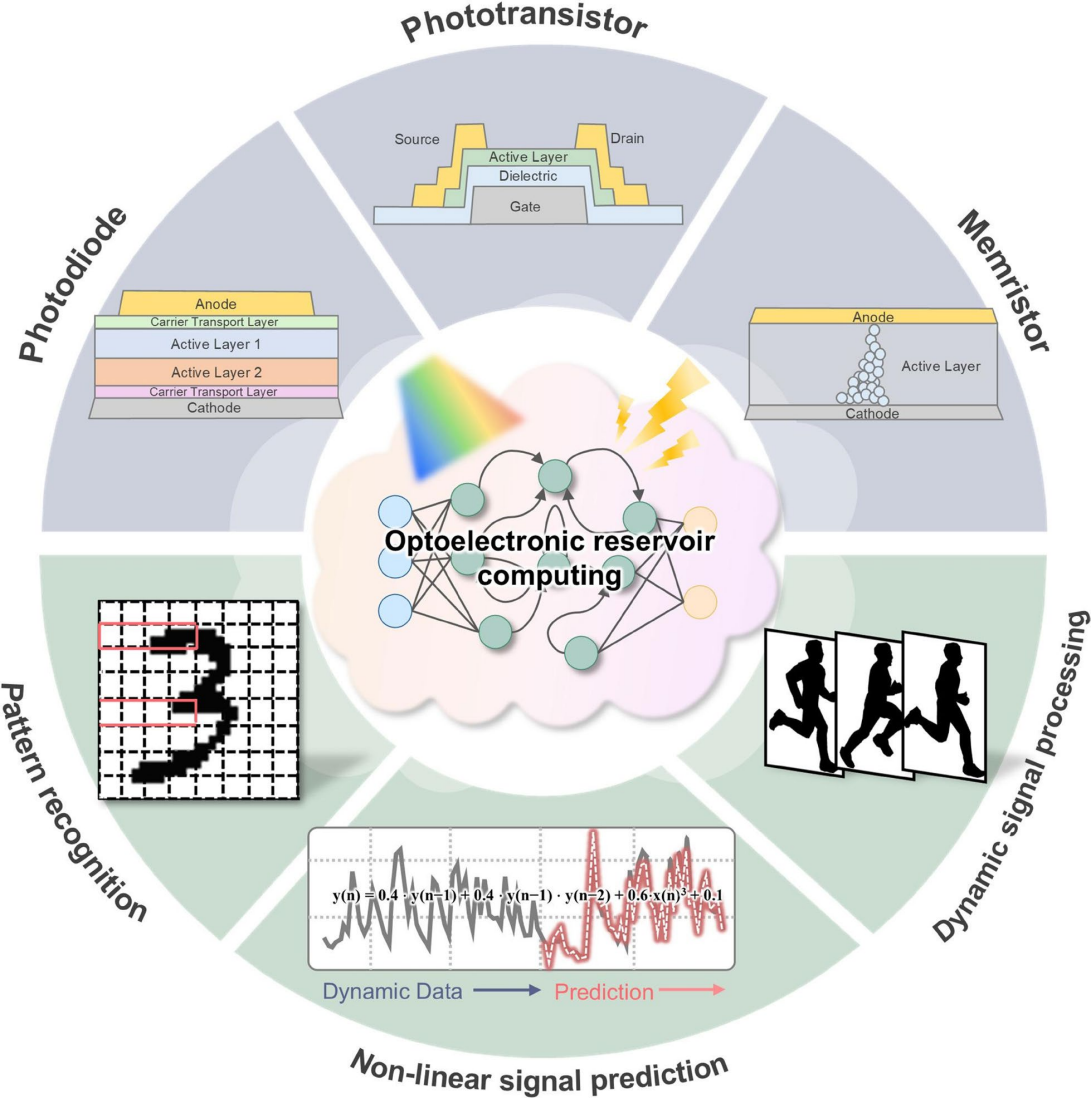

## Introduction

Artificial neural networks (ANNs) have transformed fields from image recognition and language processing to scientific discovery [1–5]. Yet, as these networks grow deeper and more complex, they demand enormous computing power, leading to high energy use, longer processing times, and difficulty scaling hardware. Current AI systems mainly run on power-hungry digital processors that constantly move large amounts of data between memory and logic, making them far less efficient than the human brain. This gap in efficiency is becoming a serious obstacle for deploying intelligent systems in edge environments, where space and power are limited. To address this, researchers are exploring ways to implement neural network functions directly in hardware, using the natural physical dynamics of devices and materials to perform computation [6–11]. By letting the device itself process information, rather than running everything in software, data can be handled in parallel, with less movement and much lower power consumption. These “in-memory” [12–14] and “in-sensor” [14–17] computing approaches, along with broader on-device computing strategies [18–23], are especially promising for real-time, always-on applications such as autonomous driving, wearable health monitoring, and distributed sensor networks, where processing must occur locally with minimal latency and power consumption.

**Fig. 1:**
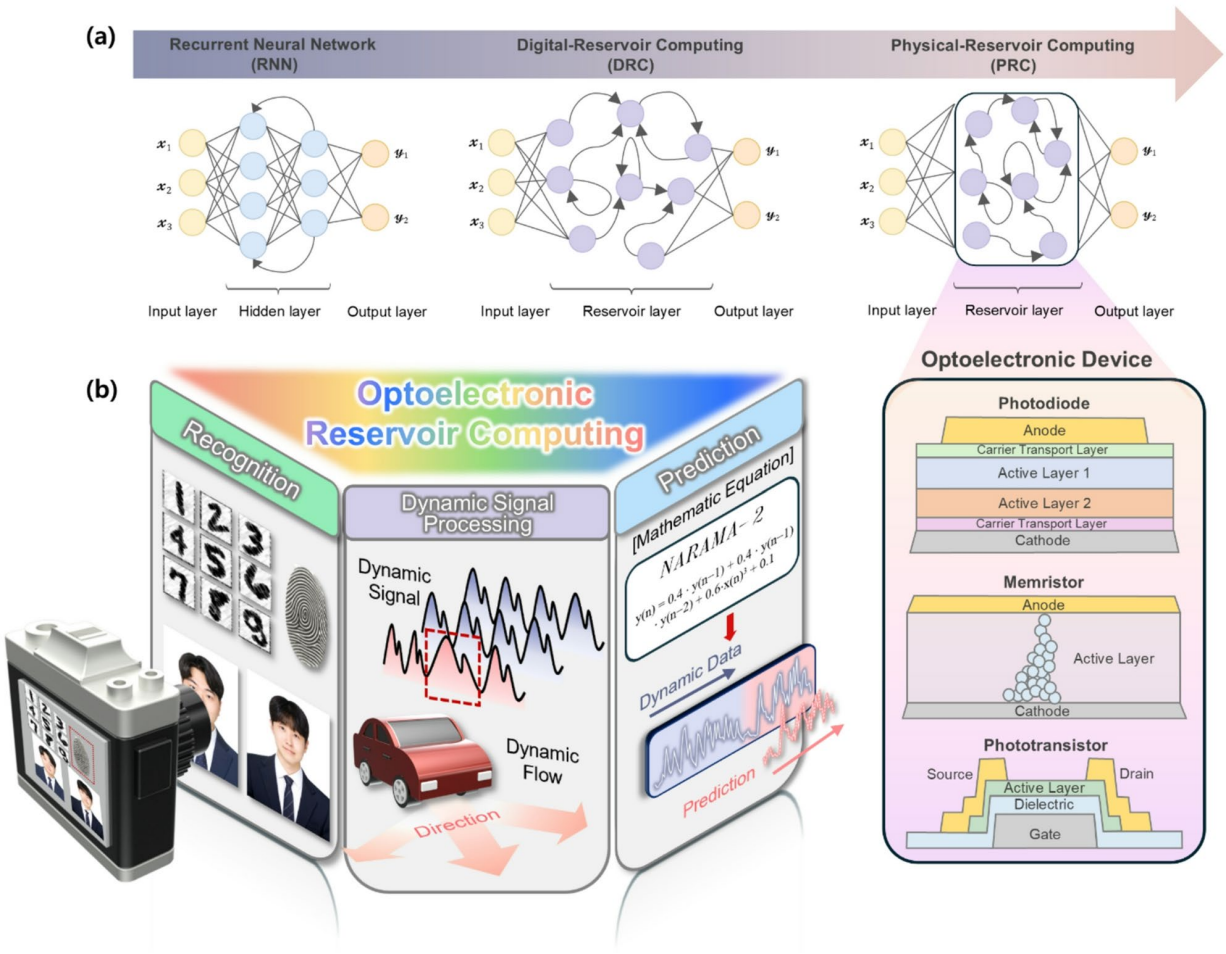
**a** Schematic illustration of the evolution from RNN to DRC and PRC. **b** The left panel shows representative applications of ORC, including image recognition, dynamic signal processing, and time-series prediction. The right panel depicts optoelectronic device implementations of PRC, such as photodiodes, memristors, and phototransistors, which provide the physical reservoir layer enabling real-time computation with enhanced speed, parallelism, and energy efficiency

Within this landscape, reservoir computing (RC) has emerged as a compelling approach for hardware-friendly neural processing. Rather than conventional recurrent neural networks (RNN) [24–27], RC fixes the internal network connections and trains only the readout layer, drastically reducing training cost and complexity [28–30]. The reservoir’s rich nonlinear dynamics and temporal memory are harnessed directly from the physical system, allowing fast adaptation to new tasks without the heavy overhead of retraining deep layers. While RNNs provide strong computational capability through trainable recurrent connections, they suffer from high training cost and stability issues. Digital-reservoir computing (DRC) alleviates these challenges by fixing the internal reservoir and training only the output layer, significantly reducing the training burden while preserving nonlinear dynamics. However, DRC implementations remain limited by digital simulations, where the richness of the reservoir states is constrained by algorithmic design and sequential computation. Physical reservoir computing (PRC), in contrast, exploits the intrinsic dynamics of physical systems to generate high-dimensional and diverse reservoir states in real time, offering richer nonlinearity, longer memory depth, and lower energy consumption. This progression from RNN to DRC and ultimately to PRC illustrates the shift toward more efficient and scalable neuromorphic computing frameworks (Fig. 1a).

A key advantage of RC is that it does not require precise control over every internal connection or weight. In other architectures, diversity and complexity in the reservoir’s response can be a drawback, but in RC they become valuable resources that broaden the system’s computational feature space [31–33]. As a simple example, RC is like tossing a pebble into a pond: ripples form, overlap, and create unique patterns. Rather than controlling them, the appropriate task is to simply observe and learn to interpret these patterns. This makes RC particularly suitable for time-series prediction [34–36], pattern recognition [37–39], and other applications where both nonlinearity and memory depth are critical [40–42].

To fully exploit these principles, hardware used for RC should provide rich nonlinear responses [43], and tunable temporal dynamics [44]. It is particularly desirable to have devices that can directly couple sensing and computation, operate with low power, and respond to a variety of input modalities. Meeting these requirements has driven active research into novel device concepts and materials including transition metal dichalcogenides [45–48], oxides [49, 50], perovskites [51], and organic semiconductors [52–59], especially those that can integrate multiple functions into a compact architecture.

Motivated by the goal of realizing device-based RC with rich nonlinearity and temporal dynamics, this review focuses on optoelectronic devices as physical reservoir computing. Their intrinsic light–matter interactions provide unique pathways to enhance nonlinearity, memory capacity, and node diversity. Light can carry information in wavelength, intensity, and pulse duration, stimulating multiple nodes in parallel with minimal delay or additional power, making optoelectronic reservoirs naturally suited for fast, and energy-efficient processing of complex temporal signals. By revisiting recent developments in photodiodes, optically modulated memristors, and phototransistors, we aim to connect device physics with reservoir dynamics and outline strategies for advancing optical RC toward practical, and scalable neuromorphic systems (Fig. 1b).

### Optoelectronic devices for reservoir computing

In reservoir computing, the synaptic connectivity between computational nodes plays a decisive role in determining the system’s memory capacity, state-space separability (computational diversity), and adaptability. Beyond software-based implementations, PRC has emerged as a major research trend, where nonlinear dynamics of real-world devices and materials are harnessed to emulate reservoir states [60–64]. A wide range of physical platforms such as memristors, spintronic devices, nanowires, and photonic structures have been explored to achieve high-speed processing, low-power operation, and hardware-level parallelism, highlighting PRC as a promising direction for energy-efficient neuromorphic systems [65–68]. Recently, there has been growing interest in optoelectronic reservoir computing (OERC) systems that directly interface photonic inputs with reservoir circuits [65]. In addition to electrical biasing, light-induced nonlinear responses enrich the input and output mapping. This provides higher-order nonlinearities and additional degrees of freedom for processing signals with strong temporal dependencies. Furthermore, the integration of optical and electrical stimuli allows tuning of device-level parameters, such as synaptic weight magnitude and sign, decay time constants, and threshold levels, thus enabling artificial synaptic plasticity [69]. While conventional RC fixes the internal reservoir connections and trains only the readout layer, OERC exploits the intrinsic device dynamics to govern the reservoir state evolution, enabling low-training-cost, real-time information processing [70].

### Non-linear synaptic response mechanism of optoelectronic devices

**Fig. 2:**
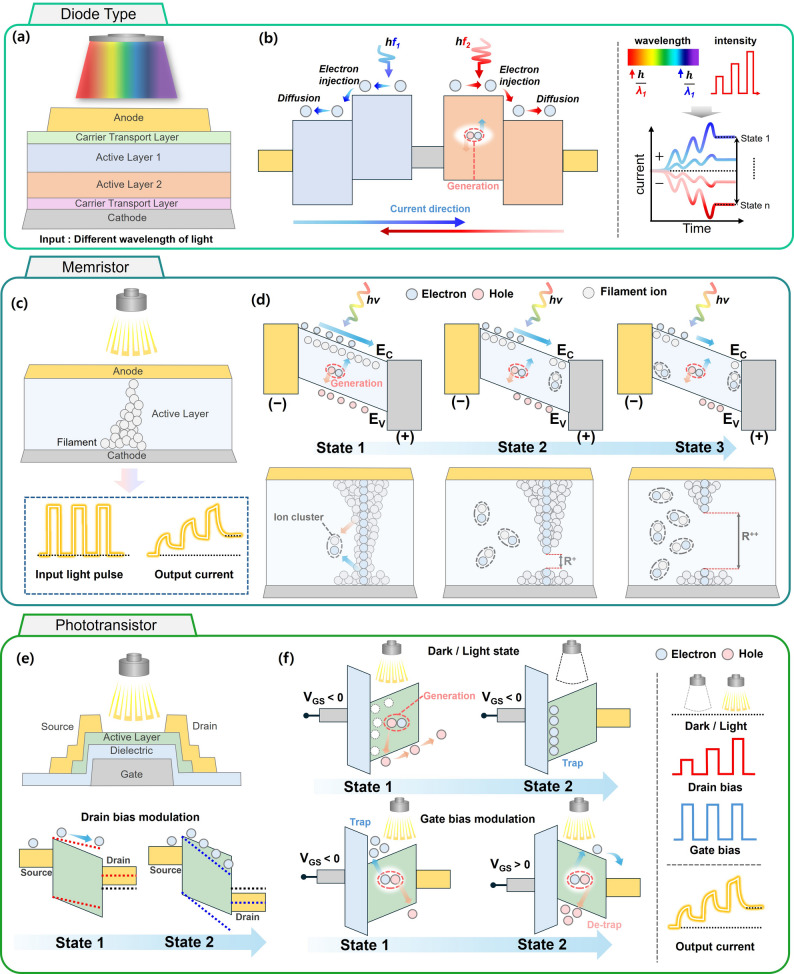
Representative structures and operating mechanisms of optoelectronic devices for physical reservoir computing. **a** Diode-type photodetector with dual absorber layers, where incident light modulates the carrier transport direction, producing distinct current outputs. **b** Carrier energy band diagram illustrating light-induced electron–hole generation and transport in the diode structure, where the current direction and electronic states are determined by the wavelength and intensity of the incident light. **c** Memristor with an active layer containing conductive filaments. **d** trap and de-trapping energy band diagrams, illustrating electron–hole pair generation by photo-carriers and their modulation under illumination and applied drain and gate voltages

OERC devices can be broadly categorized into three types. First, two-terminal photodiodes can be engineered with heterojunction interfaces, asymmetric contacts, or dual-absorber architectures to achieve wavelength and intensity dependent carrier transport modulation (Fig. 2a). In the dual-absorber configuration, distinct photoactive layers (absorber 1 and absorber 2) possess different bandgaps and carrier mobilities, enabling selective activation of electron and hole transport pathways depending on the incident light spectrum [71]. By designing the energy band alignment and carrier collection geometry, the device can not only modulate the photocurrent magnitude but also reverse its polarity in response to different optical stimuli (Fig. 2b).

Such polarity switching arises when photon absorption in different layers generates carrier populations with opposing drift directions, leading to a sign inversion in the net photocurrent [72]. The memory characteristics observed in photodiodes after optical stimulation arise from the trapping and gradual release of photogenerated carriers, as well as their delayed recombination processes. This fading memory behavior enables short-term temporal information processing without the need for long-term storage. These mechanisms allow the same device to emulate excitatory and inhibitory synaptic weights purely through optical control, without requiring electrical polarity reversal. Furthermore, the differential photocurrent between two optical states can be engineered to be highly sensitive to variations in intensity, wavelength, or modulation timing, providing a rich encoding space for reservoir computing. In an OERC system, this bidirectional current capability enables multi-channel spectral encoding and sign-reversible weight mapping at the input stage, expanding the reservoir’s feature space without additional circuit complexity. Such devices are particularly suited for tasks requiring spectral selectivity, multi-class pattern recognition, or combined spatial and spectral processing, as the photodiode’s nonlinear spectral response naturally enriches the reservoir states.

Second, two-terminal memristors exhibit resistive switching through the formation and rupture of ionic filaments or charge-trapping states, typically under electrical bias. Two-terminal memristors integrated with optical modulation offer an attractive platform for reservoir computing, as they combine resistive switching capabilities with photonic tunability (Fig. 2c). In these devices, the active layer supports both ionic filament formation/rupture and charge trapping/de-trapping processes, which can be electrically triggered and optically assisted. The optical input not only provides an additional degree of control over the conductance states but also enriches the device’s nonlinear dynamics key for enhancing reservoir state diversity.

Figure 2d illustrates the energy band diagrams and carrier transport mechanisms for three representative resistive states under light illumination. In State 1, a continuous conductive filament forms within the active layer, producing a low-resistance state (LRS). Incident light generates electron-hole pairs, with electrons injected into the conduction band (E_C_) and holes moving toward the valence band (E_V_), while localized traps temporarily capture carriers. This interaction between photocarrier generation and trap states results in short-term plasticity (STP) behavior and can be exploited to implement volatile weight updates in reservoir nodes [73]. In State 2, partial filament rupture increases the resistance, creating an intermediate conductance level. Although the primary conduction path is reduced, photogenerated carriers can still modulate the conductivity by filling traps or enabling hopping conduction between localized states. This state supports multi-bit storage, allowing finer weight resolution in reservoir computing. Optical pulses in this regime can also accelerate trap filling or facilitate ion migration, enabling controlled potentiation or depression with reduced electrical power. In State 3, the filament is further disrupted or fully ruptured, resulting in a high-resistance state (HRS). Carrier transport is dominated by trap-assisted tunneling or thermally activated hopping. Even in this regime, optical stimulation can transiently lower the resistance by generating carriers, but the overall conductance remains significantly lower than that in LRS. This makes the HRS suitable for implementing reset or decay functions within a reservoir network.

By tailoring the amplitude, duration, and spectral properties of optical stimuli in combination with electrical bias, optically modulated memristors can realize programmable STP/LTP transitions, adjustable decay constants, and tunable nonlinearity. These features are essential for adapting the reservoir’s temporal memory depth and computational richness to specific tasks, making optically assisted memristors promising building blocks for photonic reservoir computing systems [74, 75].

Third, three-terminal phototransistors can realize synaptic-like responses by independently controlling gate-voltage pulses and optical pulses under a fixed drain bias [76, 77]. As shown in Fig. 2e, the three-terminal phototransistor consists of a gate electrode beneath the dielectric layer, an active semiconductor layer between the source and drain electrodes, and an optical stimulus applied directly to the active layer. This architecture enables independent modulation of electrical and optical inputs under a fixed drain bias, thereby decoupling the read and write operations. Such separation is advantageous for neuromorphic computing because it allows multidimensional control through the gate, optical illumination, and drain bias [78, 79]. Moreover, in phototransistors, the memory capacity can be tuned by adjusting the gate bias, which represents a highly advantageous feature for reservoir computing.

Figure 2f illustrates the potentiation process under light stimulation. When a gate-source voltage (*V*_GS_) is applied, photogenerated electron–hole pairs are created within the active layer. Electrons are injected into the conduction band and may become trapped at localized defect sites, while holes move toward the valence band. This trapping process extends the carrier lifetime, effectively storing optical information as a transient conductance increase. The combination of photogeneration and charge trapping results in enhanced channel conductivity, which can be retained even after the optical pulse ends, providing short-term or long-term plasticity depending on trap characteristics [80, 81]. In contrast, Fig. 2g depicts the depression process induced by a *V*_GS_ electrical pulse. The applied gate voltage modulates the channel potential to promote carrier recombination, thereby releasing trapped electrons and reducing the channel conductivity. This de-trapping and recombination process resets the synaptic weight, enabling bidirectional plasticity essential for adaptive signal processing [78]. By controlling the amplitude, duration, and sequence of optical and gate pulses, the time constants, threshold voltages, and saturation levels of the device can be finely tuned. Such control directly influences the nonlinear transformation and memory depth of the reservoir, allowing device-level optimization for specific computational tasks in optical reservoir computing. As described above, photodiodes, memristors, and phototransistors provide tunable, optically responsive device platforms whose material and structural designs allow deliberate control of reservoir dynamics, such as temporal response, nonlinear order, and noise characteristics, making them well suited for OERC applications requiring low training cost and real-time operation.

### Time-dependent multi-state implementation for reservoir computing

In recent years, researchers have focused on generating multiple states in various optical OERC systems. Multi-state generation enables a single device to provide a larger number of reservoir nodes, thereby enriching the state space while reducing the number of physical devices required [82, 83]. For example, in transistor-type OERCs, adjusting the wavelength, intensity, or pulse width of optical stimuli, or combining them with gate voltage modulation can induce distinct conduction pathways and trap charge dynamics, resulting in nonlinear and diverse output responses. Each of these responses can be treated as an independent reservoir state, allowing the implementation of multidimensional spatiotemporal dynamics within a single device [84, 85]. Compared to conventional electrically driven RCs, which rely mainly on time-multiplexing to generate virtual nodes, OERCs provide a broader parameter space and more diverse node responses. From this point onward, we present studies that demonstrate how various optical devices generate virtual nodes through different types of stimuli.

**Fig. 3:**
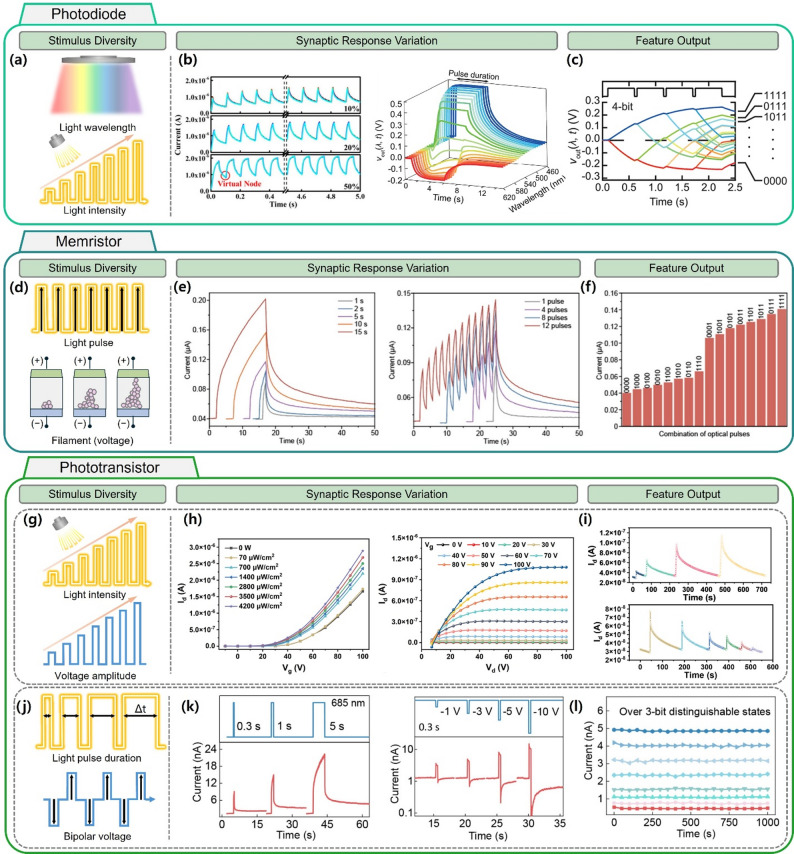
Stimulus diversity, synaptic response variation, and feature output characteristics of representative optoelectronic devices for optical reservoir computing. **a** Schematic illustration of two primary optical stimuli for generating virtual nodes in photodiode-type OERC devices: modulation of incident light intensity and variation in illumination wavelength. **b** Intensity-dependent short-term potentiation responses, in which each light intensity corresponds to a distinct virtual node (left) [79] Copyright 2024, American Chemical Society, and wavelength-selective polarity switching (right) [80] Copyright 2024, Springer Nature. **c** Mapping of multi-conductance states to digital codes, where each discrete level is assigned to a specific bit pattern [80] Copyright 2024, Springer Nature. **d** Stimulus diversity via programmable optical/electrical pulse inputs in memristor-based OERC systems. **e** Synergistic regulation of device dynamics, with electrical bias controlling filament growth and light illumination modulating carrier generation and trap occupation, leading to tunable current–voltage profiles (left) [81] Copyright 2024, American Chemical Society. and intensity-dependent transient responses (right) [81] Copyright 2024, American Chemical Society. **f** Encoding of multi-level states into binary codes, enabling a 4-bit (16-level) feature representation [81] Copyright 2024, American Chemical Society. **g** Independent or combined tuning of synaptic responses using light intensity and gate voltage amplitude in phototransistor. **h** Distinct transfer and output characteristics of a phototransistor under simultaneous modulation of incident light intensity and gate bias [84] Copyright 2023, Springer Nature. **i** Multi-level synaptic responses directly mapped to reservoir states, yielding multiple feature outputs from a single device [84] Copyright 2023, Springer Nature. **j** Additional response diversity achieved by varying light pulse duration and applying bipolar gate voltages in phototransistor. **k** Optical excitation at 685 nm combined with negative gate pulses producing pronounced potentiation and depression behaviors, thereby generating discrete and distinguishable conductance states [85] Copyright 2025, American Chemical Society. **l** Well-separated conductance states that minimize recognition errors, demonstrating the feasibility of compact, low-power OERC systems with enhanced node diversity [85] Copyright 2025, American Chemical Society

Figure 3a illustrates two primary optical stimuli for generating virtual nodes in photodiode-type OERC devices: variation in light wavelength and modulation of light intensity. In two-terminal photodiodes, these parameters directly influence photocarrier generation and transport dynamics, enabling distinct conductance states to be formed within a single device. Wan et al. demonstrated this concept using a heterojunction photodiode composed of MoO_3_/DPPT-TT/ZnO, where UV light intensity was tuned to produce multiple, well-separated conductance states (Fig. 3b, left) [86]. By incrementally adjusting the light intensity, the device exhibited reproducible short-term potentiation responses, with each intensity level corresponding to a unique virtual node in the reservoir. Komatsu et al. reported a different approach based on wavelength-selective polarity switching, employing TiO_2_/SQ2 and TiO_2_/D131 dye-sensitized structures (Fig. 3b, right) [87]. Under illumination from 300 to 750 nm, the D131-based device produced positive photocurrent, while the SQ2-based device generated negative photocurrent, enabling bidirectional weight representation. By applying sequential optical pulses at different wavelengths, a rich set of virtual nodes could be created from a single photodiode. As shown in Fig. 3c, these multi-state responses can be mapped to digital codes, where each conductance level corresponds to a specific bit pattern. Such encoding enables the realization of 4-bit (16 distinct states) or even 5-bit (32 states) resolution in a single device. A critical design consideration is ensuring that conductance levels are both clearly distinguishable and evenly spaced to minimize classification errors during computation. This multi-state, stimulus-driven encoding strategy allows photodiode-type devices to serve as compact, low-power physical reservoirs with high node diversity.

While photodiodes offer simple architecture for generating virtual nodes, implementing wavelength-dependent control in practical systems can introduce additional optical hardware overhead. In contrast, memristors retain the simplicity of a two-terminal design while enabling multi-state generation through the controllable formation and rupture of conductive filaments. As shown in Fig. 3d, both optical pulses and filament modulation via electrical bias can serve as stimuli, with the filament state directly influencing the device’s nonlinear response characteristics. Lin et al. developed an optoelectronic memristor based on a CuSCN/PbS quantum-dot heterojunction, capable of exhibiting a wide range of synaptic behaviors under combined electrical and optical excitation [88]. In their system, electrical bias controlled the degree of filament growth, while light illumination modulated carrier generation and trap occupation, enabling rich, tunable current-voltage profiles (Fig. 3e, left) and intensity-dependent transient responses (Fig. 3e, right). By precisely orchestrating sequences of optical pulses with varying amplitudes and polarities, they achieved a set of discrete, and stable conductance states. These states were mapped to binary codes, allowing the implementation of a 4-bit (16-level) feature output, as illustrated in Fig. 3f. The ability to electrically precondition the filament state and then optically fine-tune the response provides a flexible framework for virtual node generation, supporting both short-term and long-term plasticity. Such hybrid control schemes make optoelectronic memristors a promising platform for compact, low-power, and highly reconfigurable optical reservoir computing systems [89, 90].

Three-terminal phototransistor architectures provide an effective strategy for increasing controllable stimuli. Unlike two-terminal photodiodes, these architectures leverage both optical excitation and gate-voltage modulation, expanding the parameter space for virtual node generation. As shown in Fig. 3g, light intensity and gate voltage amplitude can serve as independent or combined stimuli to tailor synaptic responses. Wu et al. demonstrated this approach using a phototransistor that exhibited distinct transfer and output characteristics as a function of both incident light intensity and gate bias (Fig. 3h) [91]. Under varying optical powers, photogenerated carriers modulated the channel conductance, while the gate electric field further tuned carrier injection and trap occupancy, enabling finely resolved intermediate states. The resulting multi-level synaptic responses (Fig. 3i) could be directly mapped to reservoir states, providing multiple feature outputs from a single device. Further diversity can be introduced by varying light pulse duration and applying bipolar gate voltages, as illustrated in Fig. 3j. Li et al. implemented this strategy using an a-In_2_Se_3_/WSe_2_ heterojunction phototransistor, achieving highly tunable synaptic plasticity while reducing energy consumption [92]. As shown in Fig. 3k, optical stimulation at 685 nm combined with negative gate pulses produced pronounced potentiation and depression behaviors, effectively generating discrete, distinguishable conductance states. Such clearly separable states (Fig. 3l) are critical for minimizing recognition errors in OERC-based systems, enabling the device to function as a compact, and low-power reservoir with high node diversity [91].

Based on the above representative revisits, photodiodes, optically modulated memristors, and phototransistors individually offer unique pathways for generating diverse virtual nodes in OERC. By exploiting different excitation schemes, ranging from wavelength and intensity tuning, to electrically driven filament control with optical assistance, and to combined gate and optical modulation, these devices expand the reservoir’s state space while reducing hardware complexity. Such multi-state generation is key to enhancing nonlinearity, memory depth, and computational versatility, positioning OERC as a promising platform for next-generation neuromorphic computing.

## Optical reservoir computing training process and applications

In recent years, researchers have employed optoelectronic devices to induce various nonlinear responses, thereby forming reservoir networks [93–95]. As discussed in the previous section, multiple implementation strategies have been proposed, each aiming to enhance the efficiency and diversity of the generated reservoir networks [96, 97]. A key research focus has been on optimizing the network’s ability to produce a rich variety of dynamical states while maintaining computational efficiency. In the following section, we present representative studies that utilize such nonlinear reservoir networks, with particular emphasis on application domains where optical reservoir computing demonstrates unique advantages.

### Training process

In reservoir computing, learning is performed exclusively at the readout nodes, with the nonlinear transformation provided by the reservoir network enabling efficient and low-power training [98]. This architecture allows the system to leverage the intrinsic dynamics of the reservoir, avoiding the need for weight updates within the recurrent network itself. Such a design has been widely applied to tasks such as pattern recognition and nonlinear time-series prediction. More recently, hybrid approaches have emerged that integrate these two paradigms, either by treating dynamic image sequences as time-series signals to capture temporal correlations, or by representing time-series data as static patterns for anomaly detection [99–101].

**Fig. 4:**
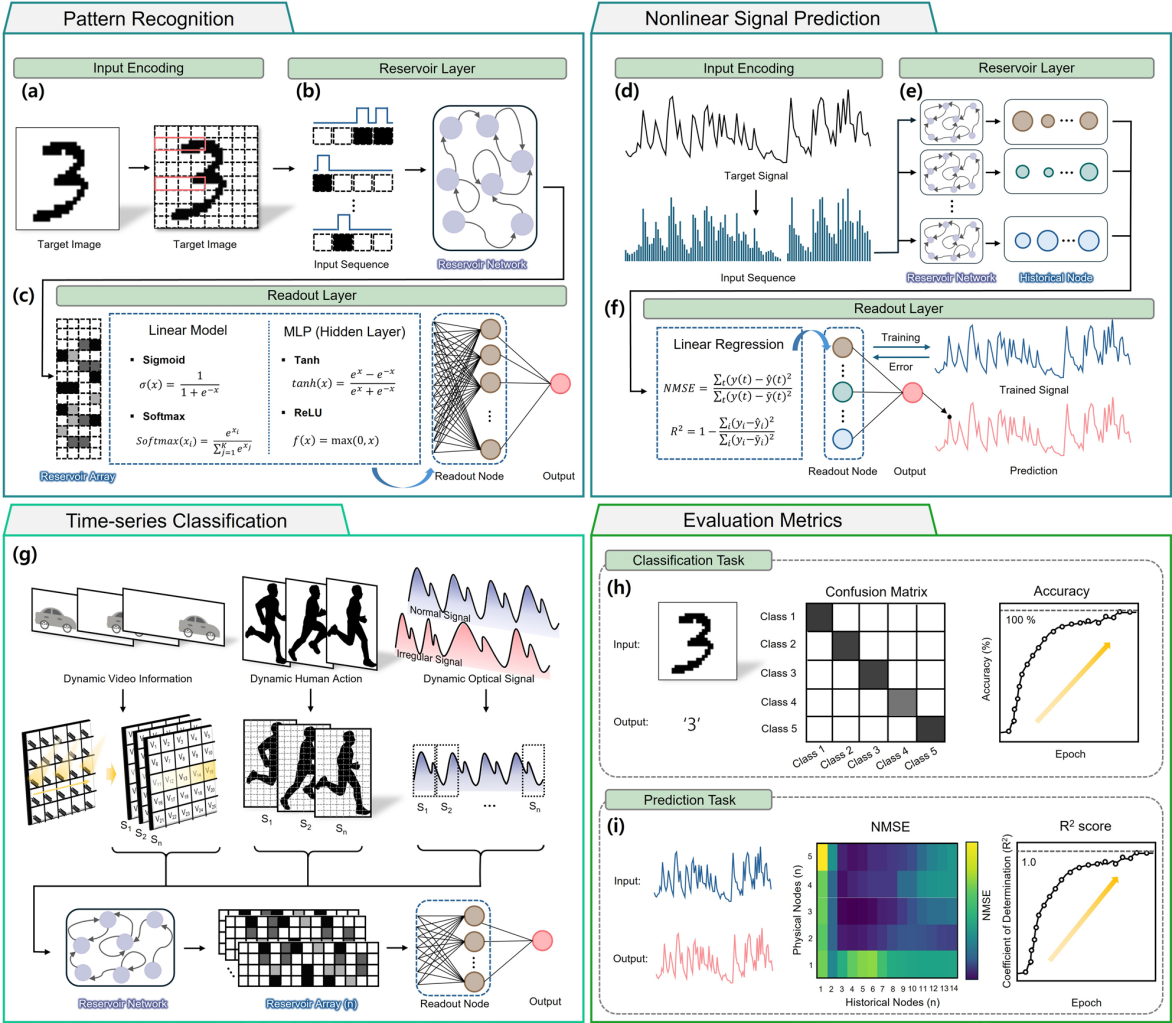
Representative tasks and evaluation metrics in RC systems. **a** Pattern recognition: An example of handwritten digit classification. The target image is converted into an input sequence through input encoding and processed by the reservoir layer. Outputs from the readout layer are computed using models such as linear regression (sigmoid, softmax) or multilayer perceptrons with nonlinear activation functions (tanh, ReLU). **b** Nonlinear signal prediction: A target time-series signal is encoded and propagated through the reservoir network comprising physical or simulated nodes. The readout layer, trained via linear regression, produces predictions with associated error metrics such as NMSE and R^2^. **c** Time-series classification: Classification of dynamic sequences, including video frames, human motion, or optical temporal signals. The encoded sequence is mapped into the reservoir layer and subsequently classified by the readout layer. **d** Evaluation metrics: For classification tasks, performance is visualized using a confusion matrix and accuracy–epoch curves. For prediction tasks, performance is quantified using NMSE maps and R^2^ score evolution over training epochs

Figure 4a illustrates the learning process for pattern recognition using PRC. Input images are encoded into an n $\:\times\:$ n pixel grid, with each pixel value sequentially injected into the OERC as an input stream. The corresponding output states from the reservoir are treated as feature vectors, effectively reducing the dimensionality of the input space. This dimensionality reduction results in fewer computations compared to conventional multilayer perceptron (MLP) image classifiers [102]. The generated reservoir states are processed at the readout node, where either simple linear model with activation functions such as sigmoid or softmax, or more complex MLP-based classifiers, are employed depending on the complexity of the image data.

As shown in Fig. 4b, nonlinear signal prediction begins with encoding a random input signal into an optical pulse sequence, and this encoded sequence is then applied to the OERC. The system generates multiple historical nodes, each reflecting different dynamical sensitivities, some tuned to capture large variations, others to detect subtle changes. This diversity in reservoir responses enhances predictive performance. The reservoir outputs (train values) are compared to the target signal, often a second-order nonlinear function, using metrics such as the normalized mean square error (*NMSE*) and the coefficient of determination (*R*^2^), defined respectively as [30, 103]:

1 $$\:NMSE\:=\:\frac{\sum\:_{i\:=\:1}^{N}{({y}_{i}\:-\:{{\hat{y}}}_{i})}^{2}}{\sum\:_{i\:=\:1}^{N}{({y}_{i}\:-\:{{\bar{y}}}_{i})}^{2}}$$

2 $$\:{R}^{2}\:=\:1\:-\:\frac{\sum\:_{i\:=\:1}^{N}{({y}_{i}\:-\:{{\hat{y}}}_{i})}^{2}}{\sum\:_{i\:=\:1}^{N}{({y}_{i}\:-\:{{\bar{y}}}_{i})}^{2}}$$

where $\:{y}_{i}$ is the target value, $\:{{\hat{y}}}_{i}$ is the predicted value, ${{\bar{y}}}_{i}$ is the mean of the target values. The readout layer, typically implemented as a linear regression model, is trained using these error metrics as feedback weights. The target signal is obtained by applying a nonlinear transformation to the random input, and this nonlinear mapping can be emulated by the OERC system. Because the reservoir network provides rich nonlinear transformations, even a linear readout layer can achieve high predictive accuracy [104].

A more advanced RC application combines the concepts of pattern recognition and time-series prediction (Fig. 4c). In such cases, dynamic data such as video sequences or human motion are encoded into multiple image frames (S_1_, S_2_, …, S_n_) that preserve temporal dependencies. Alternatively, time-series signals can be segmented into temporal snapshots and encoded as static patterns. Different from conventional RC systems that operate on a single reservoir array, these hybrid approaches train multiple reservoir arrays in parallel, enabling the system to recognize complex spatiotemporal structures.

The performance of reservoir computing systems depends on several factors, including the choice of input encoding, reservoir topology, and the number of physical and historical nodes [105–107]. For classification tasks, the confusion matrix and the evolution of accuracy over training epochs are common evaluation tools. In time-series prediction, regression-based metrics such as NMSE and R^2^ are used to quantify predictive accuracy. Optimizing the number of physical and historical nodes is critical for achieving the best trade-off between computational cost and prediction performance (Fig. 4d).

### Pattern recognition

**Fig. 5:**
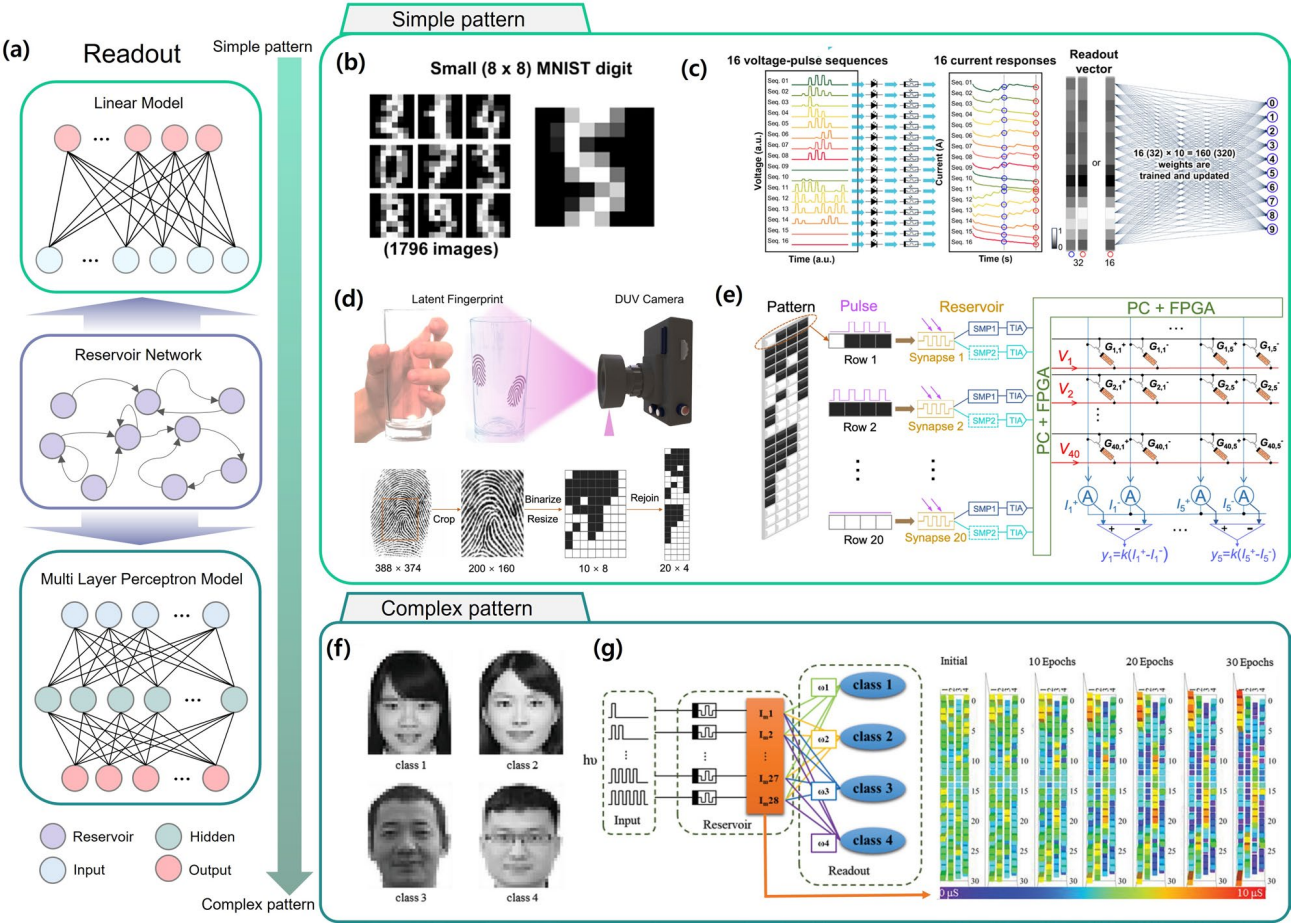
**a** Readout architectures: Linear model for simple pattern recognition, and Reservoir Network and Multi-Layer Perceptron model for complex pattern recognition. Input, hidden, output, and reservoir nodes are visually distinguished. **b** Schematic of the processing of small (8 × 8 pixels) images of handwritten digit into light pulses [104] Copyright 2024, American Chemical Society. **c** taking values from the current responses as readout values, and training the connections between readout values and output nodes [104] Copyright 2024, American Chemical Society. **d** Preprocessing method of the fingerprint images, including cropping, compressing, binarizing, and rejoining [104] Copyright 2022, Springer Nature. **e** Schematic of the proposed fully-hardware photoelectronic RC system for in-sensor fingerprint recognition, including photo-synapse reservoir layer which generates feature outputs, and memristor readout layer which performs network training [105] Copyright 2022, Springer Nature. **f** Four ID photos were chosen for face recognition and preloaded to 980 pixels in 28 × 35 size [107] Copyright 2022, Wiley-VCH. **g** Schematic of the RC system with optical pulse sequences as the inputs, the memristor reservoir, and a readout network, and weights of memristors in 28 × 4 array are updated during each training epoch [107] Copyright 2022, Wiley-VCH

Recent studies have explored strategies to achieve pattern recognition in RC with reduced computational cost [108, 109]. As discussed earlier, optoelectronic devices can be configured to represent n-bit inputs, extending beyond binary encoding, thereby enabling lower-power computation compared to conventional artificial neural network (ANN)-based training schemes. For simple images such as handwritten digits, classification can be accomplished using a single-layer linear readout model. However, as the pattern complexity increases (e.g., human face recognition), a MLP model becomes necessary to capture higher-order features (Fig. 5a) [110].

As a representative report, Jo et al. demonstrated handwritten digit recognition using a photonic synapse device implemented as a OERC system, driven by optical pulse signals (Fig. 5b) [111]. In their approach, the voltage pulses applied to the LED served as the operating stimulus. While a conventional 8 $\:\times\:$ 8 encoded handwritten image requires 64 sequential inputs The use of a 4-pulse-driven or 8-pulse-driven photonic synapse RC allowed classification with only 16 or 32 input sequences. The conductance values obtained after four optical pulses formed the readout vector, enabling low-power classification. The schematic of the input encoding process is shown in Fig. 5c. This approach achieved an accuracy of 87.59%, which is lower than the >90% typically achieved with ANN-based methods, but significantly reduced the training cost by mapping the original inputs to a smaller output vector.

To handle more complex image patterns, recent work has investigated methods to induce a greater variety of nonlinear responses within a single OERC device, thus increasing recognition accuracy while minimizing computational load. Zhang et al. fabricated an amorphous GaO_x_ (a-GaO_x_) optoelectronic synapse, integrated with a memristor array, to implement a latent fingerprint recognition system [112]. As shown in Fig. 5d, images were preprocessed through cropping, resizing, and binary pixel conversion before being injected into the OERC. To provide richer feature information, the device’s output current was sampled at different time points, generating two distinct feature sets (SMP1 and SMP2). This dual-feature-output strategy, illustrated in Fig. 5e, enabled the extraction of multiple features from a single device. The OERC outputs were then fed into the memristor array, which acted as a fully connected readout network. The classification network, trained using MATLAB with a Softmax output function and logistic regression, successfully classified high-dimensional latent fingerprint images, demonstrating the practical viability of PRC systems for security-related applications.

For even higher-dimensional patterns, such as real facial images containing grayscale and shading information, more extensive computation is required [113]. Lao et al. proposed a biomimetic approach inspired by the human visual system to classify four classes of human face images using the low-cost training advantages of RC (Fig. 5f) [114]. Their OERC utilized a self-powered memristive device composed of Au/P(VDF-TrFE)/Cs_2_AgBiBr_6_/ITO, enhancing energy efficiency. To simplify color information, input images were converted to grayscale and quantized into six intensity levels (0 to 5). As shown in Fig. 5g, the system generated and trained a 28 × 4 reservoir array for classification. While simple patterns could be learned using a single-layer linear model, the complexity of the reservoir array required training with an MLP structure containing hidden layers. This method achieved a remarkable classification accuracy of 99.97% across four classes. Compared to conventional ANN structures that require training of 28 $\:\times\:$ 35 $\:\times\:$ n parameters, the inherent nonlinearity of OERC enabled successful facial image recognition with a significantly smaller network. Furthermore, the Cs_2_AgBiBr_6_-based RC system operated in a zero-bias photovoltaic mode, simultaneously reducing both energy consumption and training cost.

### Non-linear time-series system prediction

While RC offers the advantages of reduced computation and low training cost, its classification accuracy often remains lower than that of conventional ANNs. However, for time dependent data such as nonlinear dynamical signals, the fact that ANNs produce outputs immediately after receiving inputs makes them inherently less suited to capture long term temporal dependencies [115]. Nonlinear dynamical systems are characterized by complex interactions among state variables, where both nonlinearity and temporal memory are essential. PRC provides a natural solution, as the reservoir network simultaneously offers nonlinear transformation and inherent time-delay dynamics, enabling effective mapping of such signals [116]. Although reports on nonlinear dynamical prediction using OERC remain limited, several recent studies highlight promising directions.

**Fig. 6:**
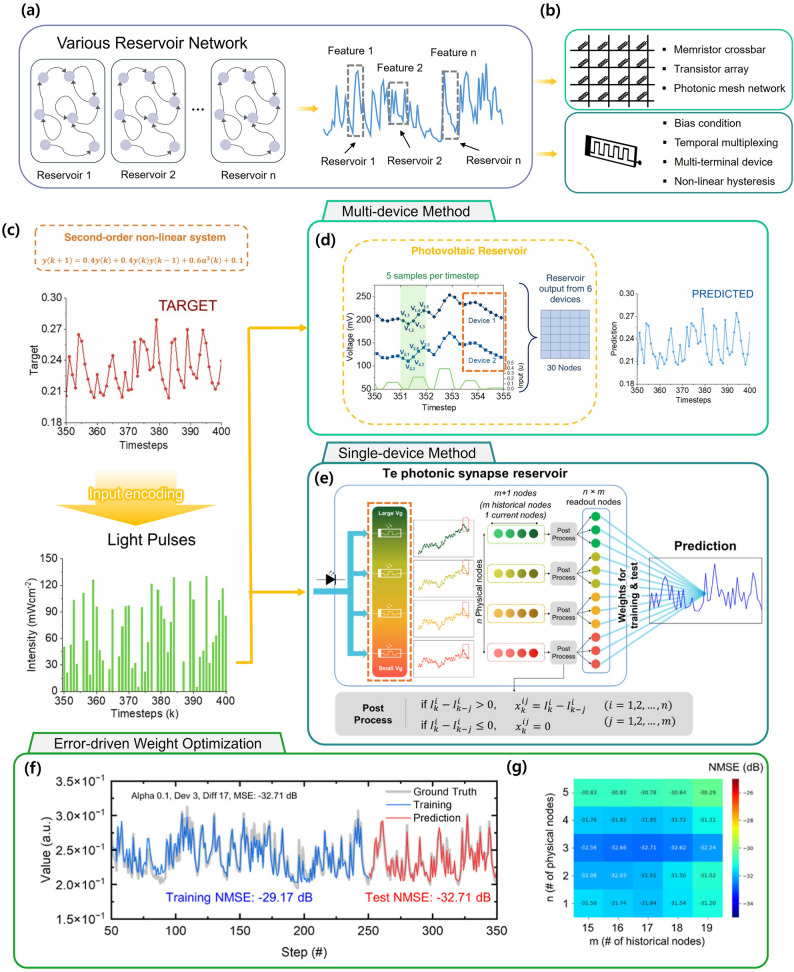
Optical reservoir computing approaches for nonlinear signal prediction using multi-device and single-device methods. **a** nonlinear dynamical signals contain diverse features, and using multiple reservoirs that are sensitive to either current or past states improves learning speed and accuracy. **b** In the multi-device approach, different physical devices, each with distinct response characteristics, are treated as separate reservoir networks to collectively form the reservoir state space. In contrast, single-device methods induce multiple nonlinear responses within a single device by tuning parameters such as bias voltage, hysteresis, or multi-terminal configurations. **c** The time-domain nonlinear target signal was encoded into a light pulse sequence and fed into the OERC [110] Copyright 2024, Elsevier. **d** Fig. 6d demonstrates that even identical encoded inputs produced different reservoir outputs across devices, indicating that device-specific nonlinearities contribute to a richer state space. Using outputs from six devices, a 6 $\:\times\:$ 5 reservoir state matrix was generated for training. Linear regression was then applied to the readout nodes, achieving a NMSE of 0.0281, demonstrating high prediction accuracy [110] Copyright 2024, Elsevier. **e** photonic synapse and modulated its gate voltage to generate multiple reservoir networks from a single physical device [104] Copyright 2024, American Chemical Society. **f** Plots of ground truth that optimizing the readout configuration is crucial for maximizing predictive performance [104] Copyright 2024, American Chemical Society. **g** Change of the normalized mean squared error (NMSE) for different choices of n and m [104] Copyright 2024, American Chemical Society

A key requirement for accurate learning and prediction of nonlinear dynamics is the implementation of reservoir networks with diverse response characteristics. As illustrated in Fig. 6a, nonlinear dynamical signals contain diverse features, and using multiple reservoirs that are sensitive to either current or past states improves learning speed and accuracy. Two main approaches exist for achieving such diversity: multi-device and single-device methods. In the multi-device approach, different physical devices, each with distinct response characteristics, are treated as separate reservoir networks to collectively form the reservoir state space. In contrast, single-device methods induce multiple nonlinear responses within a single device by tuning parameters such as bias voltage, hysteresis, or multi-terminal configurations (Fig. 6b) [108, 109].

Sharma et al. employed a multi-device method to construct several reservoir networks and demonstrated prediction of the second-order nonlinear NARMA2 benchmark task, a widely used test in RC for evaluating nonlinear memory capacity [117]. The NARMA2 task involves predicting future time-series values based on past observations, described by:

3 $$\:y(k+1)\:=\:0.4\cdot\:y\left(k\right)\:+\:0.4\cdot\:y\left(k\right)\cdot\:y(k-1)\:+\:0.6{\cdot\:u}^{3}\left(k\right)\:+\:0.1$$

where *y*(*k*) and *u*(*k*) are the target output random input, respectively. As shown in Fig. 6c, the time-domain nonlinear target signal was encoded into a light pulse sequence and fed into the OERC. Figure 6d demonstrates that even identically encoded inputs produced different reservoir outputs across devices, indicating that device-specific nonlinearities contribute to a richer state space. Using outputs from six devices, a 6 $\:\times\:$ 5 reservoir state matrix was generated for training. Linear regression was then applied to the readout nodes, achieving a NMSE of 0.0281, demonstrating high prediction accuracy.

In a single-device implementation, Jo et al. utilized a Te-based photonic synapse and modulated its gate voltage to generate multiple reservoir networks from a single physical device [111]. This variation in gate bias induced distinct nonlinear responses, expanding the reservoir state diversity (Fig. 6e). Ridge regression was employed to train the connections between the readout and output nodes for the same NARMA2 prediction task. Such an approach not only reduces the total number of devices required but also opens opportunities for creating even richer reservoir states by tuning other device parameters.

An important consideration in this context is the careful optimization of readout nodes. The number of readout nodes is typically determined by the product of the number of physical nodes (n) and the number of historical nodes (m). A physical node refers to a distinct reservoir network, while a historical node corresponds to values obtained from a single reservoir network at different sampling times. Incorporating historical nodes enables flexible weighting between present and past information. As illustrated in Fig. 6f, optimizing the readout configuration is crucial for maximizing predictive performance. Figure 6g presents an optimization map, showing that the lowest NMSE of $\:-$32.71 dB was achieved with three physical nodes and seventeen historical nodes. Interestingly, excessive inclusion of past information can degrade performance, underscoring the importance of balancing temporal depth and node diversity. Multiple strategies for optimizing readout node configurations have been reported to further enhance nonlinear prediction performance [118, 119].

### Dynamic signal processing

In the digital era, the world is continuously observed and recorded through video. Due to the large amount of information contained in video data, intermediate backups or selective data deletion are often required. RC can extract and process only the most essential features, enabling efficient recognition and learning of dynamic information [120]. This section highlights several studies that demonstrate the applicability of OERC in real-life scenarios.

**Fig. 7:**
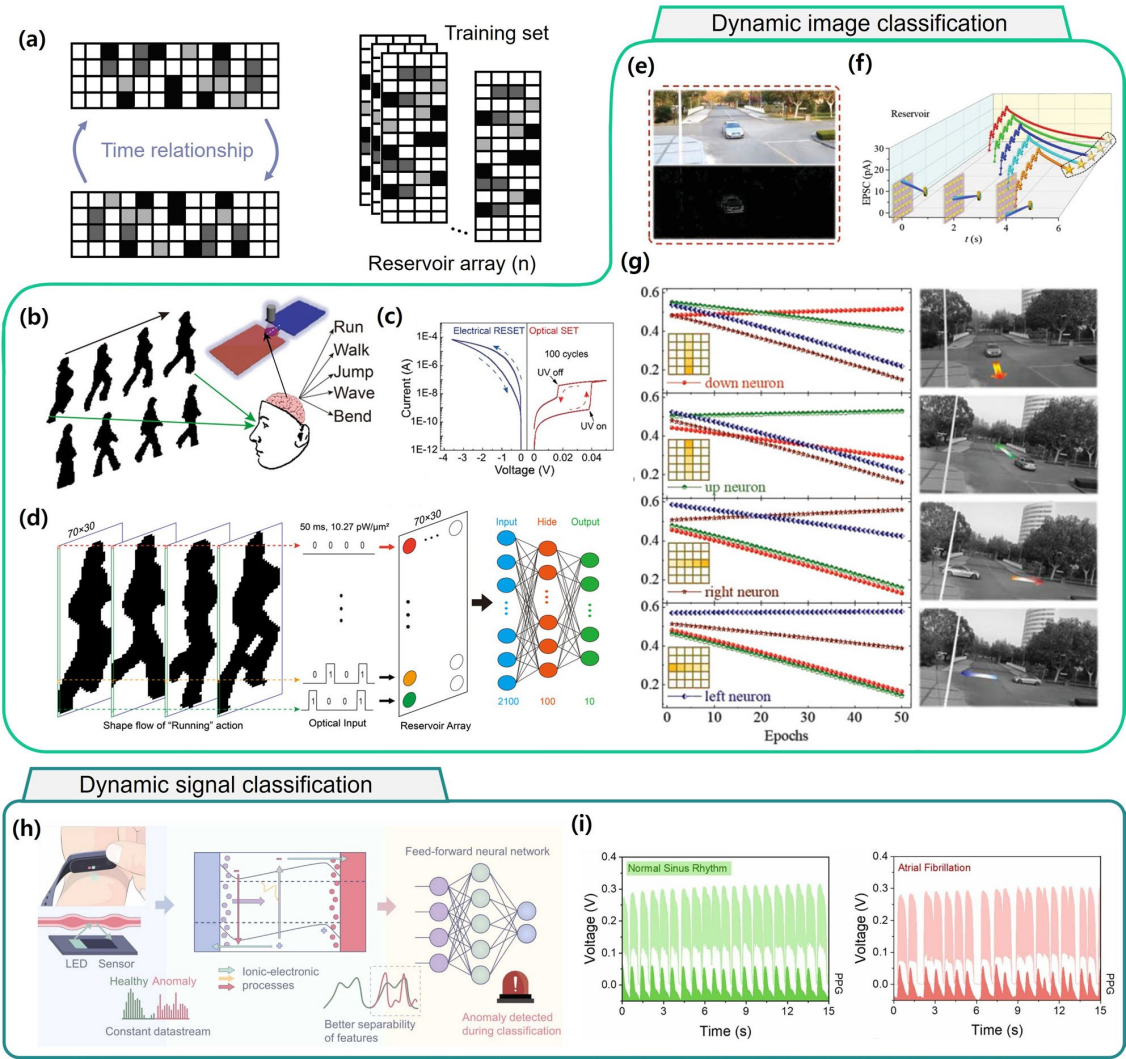
Optical reservoir computing demonstrations for dynamic image and signal classification. Dynamic image classification: **a** encoding of time relationships in image sequences into training sets. **b** schematic of human action recognition tasks with classes such as run, walk, jump, wave, and bend [114] Copyright 2022, Wiley-VCH. **c** Electrical and optical switching characteristics of the device for reservoir implementation [114] Copyright 2022, Wiley-VCH. **d** Processing of optical silhouettes of “running” motion through a reservoir array for classification [114] Copyright 2022, Wiley-VCH. Dynamic signal classification: **e** Extraction of spatial vehicle information via vehicle-pixel synchronization and conversion into optical pulse signals [107] Copyright 2022, Wiley-VCH. **f** As vehicles moved, five optical stimulations corresponding to their positions triggered excitatory postsynaptic currents (EPSCs) [107] Copyright 2022, Wiley-VCH. **g** Four movement classes, up, down, left, and right, were assigned to reservoir neurons [107] Copyright 2022, Wiley-VCH. **h** Schematic description of integration of in-sensor learning in halide perovskite solar cell to sense and compute physiological information from photo-plethysmography (PPG) [110] Copyright 2024, Elsevier. **i** Optical signal sampling at 125 Hz with five samples per detection window forming an *N* × 5 reservoir array [110] Copyright 2024, Elsevier

As illustrated in Fig. 7a, video data can be converted into multiple image frames, with temporal relationships between frames encoded for learning. Sun et al. demonstrated human action recognition using a ZnO:N/IGZO-based heterojunction device stimulated with ultraviolet light [121]. Figure 7b, schematically describes human action recognition tasks with classes such as run, walk, jump, wave, and bend. The device structure (Au/ZnO: N/IGZO/TiN) exhibited hysteresis behavior under both electrical and optical stimulation (Fig. 7c). SET and RESET operations were realized, and short-term plasticity was observed, enabling the implementation of 4-bit states for RC. In their encoding scheme (Fig. 7d), four sequential human action frames (e.g., running, walking, jumping) were overlapped. Each pixel position across these frames was then encoded as a sequence of four optical pulses. This temporal encoding mimics the way the human visual system tracks motion trajectories. The merged 70 $\:\times\:$ 30 reservoir array was connected to an MLP classifier, which successfully classified ten different actions. Across 366 randomly sampled actions, the network achieved an accuracy of 97.14%, demonstrating strong potential for bio-inspired motion recognition beyond conventional ANN methods.

As a remarkable application, Lao et al. developed an OERC-based vehicle flow recognition system integrated into a roadside camera (Fig. 7e) [114]. In this system, spatial vehicle information was extracted from video by synchronizing only the vehicle pixel data, which was then converted into optical pulse signals. These pulses were sequentially injected into a 5 $\:\times\:$ 5 optical synapse array according to the vehicle’s movement direction. As vehicles moved, five optical stimulations corresponding to their positions triggered excitatory postsynaptic currents (EPSCs) (Fig. 7f). Four movement classes, up, down, left, and right, were assigned to reservoir neurons (Fig. 7g). After 24 training epochs with weight updates, the system achieved 100% classification accuracy, showing that minimal spatial information can be sufficient for effective video-based motion tracking.

OERC has also shown promising potential in healthcare monitoring, where wearable or attachable devices must operate over long periods with simple designs and low power consumption [122, 123]. Photoplethysmography (PPG) signals, obtained from reflected light at the wrist or fingertip, require efficient and continuous optical signal processing. Sharma et al. proposed an in-sensor computing scheme using a CH_3_NH_3_PbBr_3_ perovskite-based photonic neuromorphic device for detecting atrial fibrillation (AF) from PPG signals. As shown in Fig. 7h, variations in pulse rate over time were captured and processed directly by the OERC. Optical signals were sampled at 125 Hz, five times per detection window, generating an N $\:\times\:$ 5 reservoir array (Fig. 7i). A feed-forward neural network with an MLP architecture classified signals as either normal or AF. The classification performance, measured by the area under the receiver operating characteristic curve (AUC), was 0.83, indicating reliable detection capability. This direct optical processing of PPG signals, bypassing conventional filtering and conversion steps, highlights the potential of OERC for efficient, and low-latency biomedical monitoring.

## Challenges and future direction

Reservoir computing has become an important framework for hardware-friendly neural information processing, and its research trends largely follow two representative application domains: pattern recognition and nonlinear dynamical system prediction. Pattern recognition tasks, such as handwritten digit or face classification, have provided intuitive demonstrations of RC’s potential, while dynamical prediction benchmarks like NARMA2 highlight its ability to handle time-dependent signals. These two domains have established RC as a versatile approach that can leverage device-level nonlinearity and temporal memory without incurring the training burden of conventional deep neural networks.

Within this broader trajectory, OERC has emphasized the unique role of light as both a carrier and a modulator of information. Encoding schemes based on wavelength, intensity, and temporal overlap allow optical reservoirs to generate diverse, and high-dimensional state spaces from relatively simple device architectures. Moreover, light–matter interactions enable intrinsic time constants, multi-state conductance, and spectral selectivity that are difficult to replicate purely in electronic reservoirs. This positions OERC as a natural candidate for tasks where optical inputs dominate, such as video recognition, traffic flow monitoring, or biomedical sensing [124].

Nevertheless, certain limitations remain. For static pattern recognition, deep ANNs still outperform RC in terms of accuracy, especially when very high-dimensional datasets are involved. Similarly, for precise nonlinear dynamical prediction, electronic reservoirs often offer more controllable and stable temporal dynamics than optical systems. These limitations suggest that optical RC may not replace conventional ANN or electronic RC in their strongest domains, but instead should target applications where optical encoding provides a direct advantage. Examples include continuous video streams, where motion trajectories can be encoded into temporal pulse sequences at the pixel level, and PPG monitoring, where intrinsic optical fluctuations can be processed directly without conversion overhead.

Another critical limitation of RC is its relatively slow computational speed, particularly in implementations that rely on time-multiplexing to generate virtual nodes. However, OERC offers unique pathways to alleviate this issue. First, the intrinsic parallelism of light can be harnessed to generate multiple virtual nodes simultaneously. Techniques such as wavelength-division multiplexing, polarization multiplexing, and spatial multiplexing allow the reservoir to process many channels of information in parallel, significantly reducing latency compared to purely time-sequential electrical approaches. Second, fast response in the gigahertz regime can be achieved either by employing optoelectronic devices such as photodiodes or by utilizing advanced materials such as perovskites and two-dimensional semiconductors [125–127]. By leveraging these ultrafast optical responses, reservoir nodes can update at much higher frequencies than conventional electronic systems. While such advances do not make RC a universal replacement for ANNs, they provide a practical route to high-throughput and real-time performance in domains where ANN-based solutions are inefficient or impractical.

## Conclusions

Optically driven PRC has emerged as a promising paradigm for low-power, real-time neuromorphic computation by leveraging the intrinsic nonlinear dynamics of optoelectronic devices. This review discusses the unique advantages of optically integrated reservoir computing and focuses on the application domains that leverage these strengths. Through the integration of photonic and electrical stimuli, OERC systems can enrich their state space, achieve tunable synaptic plasticity, and generate multi-state responses without adding circuit complexity. Device platforms such as photodiodes, optically modulated memristors, and phototransistors each provide unique advantages for virtual node generation. These advantages range from spectral selectivity and bidirectional weight control to hybrid electrical and optical modulation schemes that enhance nonlinearity and memory depth.

Table 1 summarizes representative optoelectronic devices used for photonic reservoir computing, highlighting their implementation strategies, operating mechanisms, advantages, limitations, and application domains. Photodiodes, with heterojunction or dual-absorber structures, offer simple fabrication, fast response, and bidirectional weight modulation through optical control, but their nonlinearity and memory depth are limited, restricting their use to relatively simple tasks such as digit or face recognition. Optically modulated memristors, incorporating absorbance layers to enable resistive switching, provide strong nonlinearity and tunable short- to long-term plasticity at low power, yet suffer from slow response and device-to-device variability, making them suitable for applications such as fingerprint recognition. In addition, memristors can be integrated into large-scale crossbar arrays, which is advantageous for scalable and high-density reservoir networks. Phototransistors, with their three-terminal architecture, allow independent electrical and optical modulation, multi-level state generation, and tunable memory depth, despite the cost of higher power consumption and more complex fabrication. These characteristics make them particularly effective for demanding spatiotemporal tasks such as vehicle flow monitoring and human action recognition.

**Table 1:** Summary of optoelectronic devices for reservoir computing

Device type	Implementation	Key modulation mechanisms	Advantages	Limitations	Application
Photodiode (2-terminal)	Heterojunction, dual-absorber	- Optical modulation	- Simple fabrication - Fast response - Bidirectional weight - Low power	- Limited nonlinearity - Limited control of memory capacity	- Digit/face recognition
Optical memristor (2-terminal)	Inserted absorbance layer	- Resistive switching - Optical modulation	- STP–LTP tunable - Strong nonlinearity - Low power	- Slow response - Variability - Endurance	- Digit/Fingerprint recognition
Phototransistor (3-terminal)	Single channel	- Gate modulation - Optical modulation - Charge trapping	- STP–LTP tunable - Read/write separation - Tunable memory depth - Multi-level states	- Complex fabrication - Slow response - High power	- Digit recognition - Vehicle flow - Action recognition

To provide a balanced comparison among different neural network architectures, Table 2 summarizes the key advantages and limitations of ANN, RNN, DRC, and the proposed OERC. Conventional ANNs are efficient for static data processing but lack temporal dynamics, whereas RNNs enable sequential learning through feedback loops at the expense of training stability and computational cost. DRC simplifies the training process by updating only the readout layer, offering moderate scalability and effective short-term memory. In contrast, the proposed OERC leverages intrinsic optoelectronic nonlinearities to achieve high-speed, low-power computation with tunable temporal responses. Although fabrication complexity and large-scale integration remain challenging, OERC demonstrates strong potential for real-time, energy-efficient reservoir computing platforms.

**Table 2:** Summary of the comparative on the advantages and limitations of ANNs, RNNs, DRC, and OERC

Category	ANN	RNN	DRC	OERC
Processing Type	Static, feed-forward computation	Sequential processing with feedback loops	Temporal via fixed reservoir	Parallel, optoelectronic feedback
Nonlinearity Source	Activation functions	Activation + recurrent feedback	Reservoir node interactions	Optical nonlinearities
Memory / Temporal Dynamics	None	Temporal memory	Short-term memory	Tunable temporal response
Training Complexity	Full backpropagation	Backpropagation through time	Only readout layer trained (simple linear regression)	Only readout layer trained
Training Efficiency	High computational cost for deep networks	High due to sequential dependency	Moderate; depends on reservoir size	High; optical domain enables low latency and energy efficiency
Scalability	High in digital systems	Limited by training time	Moderate scalability in digital hardware	High with photonic integration
Main Advantages	Simple and versatile for static data	Strong sequential data handling	Easy training, temporal processing	High bandwidth, low power, real-time analog computation
Main Limitations	No time processing	Training instability and gradient issues	Limited precision	Fabrication complexity

Recent studies highlight this strength through applications such as vehicle flow monitoring and PPG signal analysis, where OERC devices are integrated directly into sensing platforms. For example, in vehicle flow recognition, spatial trajectories extracted from video streams are temporally encoded into optical pulse sequences, allowing the reservoir to inherently preserve motion continuity without external memory modules. Similarly, in PPG-based atrial fibrillation detection, optical signals are injected directly into the reservoir array in their native form, and bypassing conventional digitization and filtering stages. These encoding strategies, whether through wavelength multiplexing, intensity modulation, or temporally overlapped pulse sequences, transform raw, high bandwidth input streams into compact yet information rich reservoir states. As a result, OERC offers both low training costs and the ability to tailor optimizations for specific applications. This makes it a promising platform for next generation embedded intelligence in dynamic and resource constrained environments.

## Data Availability

No new data were generated in this study. All relevant information has been obtained from published sources, which are cited in the manuscript.

## Abbreviations

RC: Reservoir computing
ANNs: Artificial neural networks
RNNs: Recurrent neural networks
DRC: Digital-reservoir computing
PRC: Physical reservoir computing
OERC: Optoelectronic reservoir computing
STP: Short-term plasticity
LTP: Long-term plasticity
LRS: Low-resistance state
HRS: High-resistance state
MLP: Multilayer perceptron
NMSE: Normalized mean square error
R^2^: Coefficient of determination
PPG: Photoplethysmography
AF: Atrial fibrillation
AUC: Area under the (receiver operating characteristic) curve
